# Association of University Student Gatherings With Community COVID-19 Infections Before and After the NCAA March Madness Tournament

**DOI:** 10.1001/jamanetworkopen.2021.30783

**Published:** 2021-10-25

**Authors:** Ashley L. O’Donoghue

**Affiliations:** 1 Center for Healthcare Delivery Science, Beth Israel Deaconess Medical Center, Boston, Massachusetts

## Abstract

This cross-sectional study examines the association of large celebrations at universities across the US, before and after the NCAA March Madness tournament, with COVID-19 infections in a university’s county.

## Introduction

Universities across the US are weighing various vaccination policies for the current fall semester.^1^ It is difficult to assess the risk of social gatherings on university campuses among unvaccinated students. However, the NCAA Men’s Division I Basketball Tournament (March Madness) provides an opportunity to study this risk. In mid-March 2021, when variants were spreading rapidly^2^ and vaccination rates were low among the college-aged population,^3^ 64 universities across the country competed in March Madness. By the event’s nature as a single-elimination tournament, it is difficult to predict which universities will proceed to the later rounds of the tournament. This cross-sectional study uses a difference-in-differences analysis to estimate the association of large celebrations at universities across the US with COVID-19 infections in a university’s county during a time of low vaccination rates among university students.

## Methods

 This research was classified as not human subjects research by the Beth Israel Deaconess Medical Center institutional review board; thus, informed consent was not needed in accordance with 45 CFR §46. This study follows the Strengthening the Reporting of Observational Studies in Epidemiology (STROBE) reporting guideline for cohort studies.

The primary outcome of this study is new daily COVID-19 infections in each county per 100 000 residents. These data came from the *New York Times*^4^ from January 28, 2021, to May 25, 2021, spanning from 50 days before to 50 days after the tournament. Because social gatherings often become larger later in the tournament, the day of exposure is defined as the date of the latest game a team plays in the tournament.

A difference-in-differences design was used to compare counties with universities competing in March Madness with counties within the same states that were not competing, before and after the tournament (see the eAppendix in the Supplement for details). Analyses were performed in Stata/SE statistical software version 16 (StataCorp). All *t* tests were 2-tailed, and *P* < .05 was considered significant.

## Results

Summary statistics of new daily COVID-19 infections per 100 000 before and after the tournament by county participation status are presented in the Table. The Figure displays the daily event study estimates, comparing participating and nonparticipating counties before and after the tournament. The necessary parallel pretrends assumption was satisfied and is displayed in the Figure. The daily event-study estimates first show a significant change in COVID-19 infections in participating counties 8 days after the final game of participation (13.4%; 95% CI, 0.8% to 25.9%). The estimates continued to be significant, peaking on day 24 (21.8%; 95% CI, 6.4% to 37.3%) and then decreasing until day 30 when there was no longer a significant difference from nonparticipating counties (17.1%; 95% CI, –0.6% to 34.7%). The results also hold with a control group of counties in the same states with undergraduate degree-granting universities with an enrollment of at least 200 students.

**Table.** Summary Statistics of New Daily COVID-19 Infections Before and After March Madness in Participating vs Nonparticipating Counties

Period	Infections per 100 000 residents, median (IQR), No.^a^	
	Nonparticipating counties (n = 2520)	Participating counties (n = 61)
Before tournament^b^	14.2 (0.0-29.7)	19.2 (10.9-32.7)
After tournament^c^	6.6 (0.0-16.3)	9.9 (4.1-17.0)

^a^ Table displays unadjusted medians and IQR of new daily county-level COVID-19 infections per 100 000 residents between counties with a university participating in the NCAA Men’s Division I Basketball Tournament (March Madness) and counties not participating in the tournament from January 28, 2021, to May 25, 2021.

^b^ The period before the tournament is defined as January 28, 2021, to March 17, 2021.

^c^ The period after the tournament is defined as after April 6, 2021, to May 25, 2021.

**Figure.**
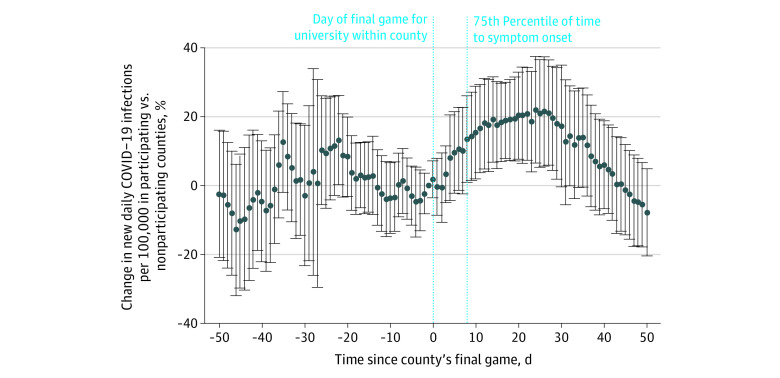
Difference in New Daily COVID-19 Infections per 100 000 in Counties With Universities Participating in March Madness vs Counties Without Universities Participating in the Tournament Graph shows difference in COVID-19 rates per 100 000 individuals in counties with a university participating in the NCAA Men’s Division I Basketball Tournament (March Madness) from January 28, 2021, to May 25, 2021, compared with the control group of nonparticipating counties within the same states. Both the participating and nonparticipating counties had decreasing COVID-19 rates during this time period. Thus, this figure displays the slowing of the decline in participating counties and brief increases in infections, compared with nonparticipating counties. The 75th percentile of time to symptom onset was 8 days. The 95% CIs are represented by the error bars, and the dots denote point estimates. Estimates come from a difference-in-differences event study model. This model includes county indicators to adjust for time-invariant differences across counties, as well as week-of-year indicators to adjust for changes in COVID-19 infections that varied across time but did not vary across counties. Robust SEs were clustered at the state level. The outcome variable was normalized with a log-transformation. The preperiod estimates are all insignificant, meaning that the parallel pretrends assumption was satisfied. Please see the eAppendix in the Supplement for more details.

## Discussion

The findings of this cross-sectional study suggest that social gatherings among unvaccinated students were associated with increased COVID-19 infections (in this scenario, slowing the previous downward trend and briefly increasing) in a university’s community beginning 8 days after the event, which corresponds with the 75th percentile of time to symptom onset.^5^ There are some limitations to this study. First, states vary in their testing and reporting of COVID-19 infections over time. Second, universities participating in March Madness may have increased surveillance testing during and after the tournament and, thus, documented more COVID-19 infections during this time than in other counties, but the data set does not stratify by whether the infections occurred at a university.

This study identifies an urgent gap in evidence on the risk of COVID-19 spread at social gatherings among university students, although the increase in transmission was brief. This increase in transmission may have been brief because of increases in the vaccination rate of university students during this time or because some students may have completed their semester before the end of the study period.

## Notes

Supplement. **eAppendix.** Supplemental Methods
