# Supplementary material for: Association of University Student Gatherings With Community COVID-19 Infections Before and After the NCAA March Madness Tournament
Source: JAMA Netw Open. 2021 Oct 25;4(10):e2130783. doi: 10.1001/jamanetworkopen.2021.30783 (PMC8546495; doi:10.1001/jamanetworkopen.2021.30783)
Supplement: Supplement. — eAppendix. Supplemental Methods [file jamanetwopen-e2130783-s001.pdf]

Supplemental Online Content

O’Donoghue AL. Association of university student gatherings with community COVID-19 infections before and after the NCAA March Madness tournament. *JAMA Netw Open*. 2021;4(10):e2130783. doi:10.1001/jamanetworkopen.2021.30783

eAppendix. Supplemental Methods

This supplemental material has been provided by the authors to give readers additional information about their work.

© 2021 O’Donoghue AL. *JAMA Network Open.*

eAppendix. Supplemental Methods

Three counties (Spokane, WA; Los Angeles, CA; and Harris, TX) had two universities competing in March Madness. For those counties, the date of the latest game for either team is used as the date of exposure. The outcome of interest was new daily COVID-19 infections at the county-level, per 100,000 residents. There were some negative values for daily infections in the New York Times data, which were re-coded as zero (less than 2% of the sample and this does not change the findings). Due to differences in day-of-week COVID-19 test processing and reporting by state, especially on weekends, the outcome measure was smoothed using a 7-day moving average and normalized with a log-transformation (as a monotonic transformation, 1 was added to the outcome before taking the logarithm to avoid the issue of log(0) being undefined). To evaluate whether a university's participation in March Madness was associated with increases in COVID-19 infections in the university's county, a difference-in-differences event study design was estimated to compare counties with universities competing in March Madness with counties that are not involved in March Madness (but are located in the same states), before and after the tournament. Because trends in new COVID-19 infections in the March Madness-participating counties are parallel to new COVID-19 infections in non-March Madness-participating counties, deviations from the trend in March Madness-participating counties in the days following the tournament are associated with the tournament. This methodological design includes county indicators to adjust for time-invariant differences across counties. It also includes week-of-year indicators to adjust for changes in COVID-19 infections that varied across time but did not vary across counties. Robust standard errors were clustered at the state level.

© 2021 O'Donoghue AL. *JAMA Network Open.*
